# SARS-CoV-2 infection and vaccine effectiveness in England (REACT-1): a series of cross-sectional random community surveys

**DOI:** 10.1016/S2213-2600(21)00542-7

**Published:** 2022-04

**Authors:** Marc Chadeau-Hyam, Haowei Wang, Oliver Eales, David Haw, Barbara Bodinier, Matthew Whitaker, Caroline E Walters, Kylie E C Ainslie, Christina Atchison, Claudio Fronterre, Peter J Diggle, Andrew J Page, Alexander J Trotter, Deborah Ashby, Wendy Barclay, Graham Taylor, Graham Cooke, Helen Ward, Ara Darzi, Steven Riley, Christl A Donnelly, Paul Elliott, COVID-19 Genomics UK consortium

**Affiliations:** a School of Public Health, Imperial College London, London, UK; b MRC Centre for Environment and Health, School of Public Health, Imperial College London, London, UK; c MRC Centre for Global infectious Disease Analysis and Jameel Institute, Imperial College London, London, UK; d Centre for Infectious Disease Control, National Institute for Public Health and the Environment, Bilthoven, Netherlands; e CHICAS, Lancaster Medical School, Lancaster University, UK and Health Data Research, Lancaster, UK; f Quadram Institute, Norwich, UK; g Department of Infectious Disease, Imperial College London, London, UK; h Imperial College Healthcare NHS Trust, London, UK; i National Institute for Health Research Imperial Biomedical Research Centre, London, UK; j Institute of Global Health Innovation, Imperial College London, London, UK; k Department of Statistics, University of Oxford, Oxford, UK; l Health Data Research (HDR) UK, Imperial College London, London, UK; m UK Dementia Research Institute, Imperial College London, London, UK

## Abstract

**Background:**

England has experienced a third wave of the COVID-19 epidemic since the end of May, 2021, coinciding with the rapid spread of the delta (B.1.617.2) variant, despite high levels of vaccination among adults. Vaccination rates (single dose) in England are lower among children aged 16–17 years and 12–15 years, whose vaccination in England commenced in August and September, 2021, respectively. We aimed to analyse the underlying dynamics driving patterns in SARS-CoV-2 prevalence during September, 2021, in England.

**Methods:**

The REal-time Assessment of Community Transmission-1 (REACT-1) study, which commenced data collection in May, 2020, involves a series of random cross-sectional surveys in the general population of England aged 5 years and older. Using RT-PCR swab positivity data from 100 527 participants with valid throat and nose swabs in round 14 of REACT-1 (Sept 9–27, 2021), we estimated community-based prevalence of SARS-CoV-2 and vaccine effectiveness against infection by combining round 14 data with data from round 13 (June 24 to July 12, 2021; n=172 862).

**Findings:**

During September, 2021, we estimated a mean RT-PCR positivity rate of 0·83% (95% CrI 0·76–0·89), with a reproduction number (R) overall of 1·03 (95% CrI 0·94–1·14). Among the 475 (62·2%) of 764 sequenced positive swabs, all were of the delta variant; 22 (4·63%; 95% CI 3·07–6·91) included the Tyr145His mutation in the spike protein associated with the AY.4 sublineage, and there was one Glu484Lys mutation. Age, region, key worker status, and household size jointly contributed to the risk of swab positivity. The highest weighted prevalence was observed among children aged 5–12 years, at 2·32% (95% CrI 1·96–2·73) and those aged 13–17 years, at 2·55% (2·11–3·08). The SARS-CoV-2 epidemic grew in those aged 5–11 years, with an R of 1·42 (95% CrI 1·18–1·68), but declined in those aged 18–54 years, with an R of 0·81 (0·68–0·97). At ages 18–64 years, the adjusted vaccine effectiveness against infection was 62·8% (95% CI 49·3–72·7) after two doses compared to unvaccinated people, for all vaccines combined, 44·8% (22·5–60·7) for the ChAdOx1 nCov-19 (Oxford–AstraZeneca) vaccine, and 71·3% (56·6–81·0) for the BNT162b2 (Pfizer–BioNTech) vaccine. In individuals aged 18 years and older, the weighted prevalence of swab positivity was 0·35% (95% CrI 0·31–0·40) if the second dose was administered up to 3 months before their swab but 0·55% (0·50–0·61) for those who received their second dose 3–6 months before their swab, compared to 1·76% (1·60–1·95) among unvaccinated individuals.

**Interpretation:**

In September, 2021, at the start of the autumn school term in England, infections were increasing exponentially in children aged 5–17 years, at a time when vaccination rates were low in this age group. In adults, compared to those who received their second dose less than 3 months ago, the higher prevalence of swab positivity at 3–6 months following two doses of the COVID-19 vaccine suggests an increased risk of breakthrough infections during this period. The vaccination programme needs to reach children as well as unvaccinated and partially vaccinated adults to reduce SARS-CoV-2 transmission and associated disruptions to work and education.

**Funding:**

Department of Health and Social Care, England.

## Introduction

The UK has experienced one of the highest SARS-CoV-2 infection and COVID-19 fatality rates in Europe since the start of the pandemic. However, the UK was also one of the first countries to implement a national vaccination programme, starting in December, 2020, with rollout to the population initially targeted at those most at risk, including older people (starting with those aged ≥80 years), health and social care workers, and people with specified health conditions.^1^

The rapid spread of the delta (B.1.617.2) variant in England from May, 2021, coincided with a third wave of infections,^2^ and the prevalence of infections remained high into the summer and beyond. During August, 2021, the incidence of RT-PCR-confirmed cases of SARS-CoV-2 infection presenting to the national testing programme in England (Pillar 2) increased gradually by more than 10% overall.

**Research in context**

**Evidence before this study**

A search on PubMed for studies with the keywords “vaccine effectiveness”, “SARS-CoV-2”, and “Delta” in the title or abstract without any language or other restrictions, identified 24 results (with no duplicates). All 24 studies identified were evaluated. Outcomes varied between studies, including all infections, symptomatic infections, high viral loads, and severe cases of COVID-19. Some studies focused on particularly vulnerable or highly exposed populations such as care home residents and frontline workers. A second search of PubMed for studies with the keywords “incidence”, “prevalence”, “SARS-CoV-2”, and “September 2021” in the title or abstract, without any language or other restrictions, identified three results (with no duplicates). None was related to the incidence or prevalence of SARS-CoV-2 infection in September, 2021, with two studies being published earlier in 2021, and the third only mentioned deaths due to COVID-19 by September, 2021, worldwide.

**Added value of this study**

We analysed data from throat and nose swabs collected at home by a randomly selected sample of residents of England, aged 5 years and older, obtained during round 14 (Sept 9–27, 2021) of the REal-time Assessment of Community Transmission-1 (REACT-1) study. We estimated a weighted prevalence of SARS-CoV-2 of 0·83% (95% CrI 0·76–0·89) in England in September, 2021, which was higher than that estimated in July, 2021 (0·63%, 95% CrI 0·57–0·69]). We estimated that 4·63% of infections were related to variants carrying the Tyr145His mutation in the spike protein. We estimated SARS-CoV-2 epidemic growth rates in the community and prevalence by demographic characteristics, including vaccination status, age, ethnicity, deprivation status, and region. We found higher SARS-CoV-2 prevalence and a growing epidemic in children aged 17 years and younger, in whom vaccine coverage rates were low, while the epidemic appeared to decrease in those aged 18–64 years in September, 2021. Our results also indicated regional heterogeneity, with an increasing epidemic in London and the East Midlands. At ages 18–64 years, we estimated vaccine effectiveness against infection of 62·8% (95% CI 49·3–72·7) for those who had received two vaccine doses (across all vaccine types). At ages 18 years and older, we found a higher prevalence of swab positivity in those who had received their second dose of the vaccine 3–6 months before sample collection than in those who received their second dose within 3 months of swabbing.

**Implications of all the available evidence**

Population surveys provide a robust basis for characterisation of transmission dynamics nationally and within subpopulations. The findings of heightened transmission of SARS-CoV-2 in first few weeks of the school year in England, combined with evidence that vaccinated individuals are more likely to experience breakthrough infections more than 3 months after the second vaccine dose, make a compelling case that vaccination of older school-aged children (ie, those aged 12–17 years) and booster doses for adults aged 18 years and older should help limit the spread of SARS-CoV-2 in the population and the associated disruptions to work and education.

In September, 2021, the rollout of the national vaccination programme against COVID-19 in England was extended to offer a booster (third) dose, at least 6 months following the second dose, to health and social care workers, all individuals older than 50 years, and younger people at risk. This extension also included vaccination of older school-aged children (aged ≥12 years), who were originally offered a single vaccine dose across the UK. However, by mid-September, 2021, the number of people receiving first vaccination doses dropped to its lowest level (just under 21 000 doses per day on average in the UK) since at least mid-January, 2021 (the earliest data publicly available). Nonetheless, as of Sept 26, 2021, almost 90% of individuals aged 18 years and older in England had received their first dose, more than 83% had received their second dose, and 13% had received a third vaccine dose. Among children aged 12–17 years, 8·9% had received a single dose. Approximately 53% of adults were vaccinated with the ChAdOx1 nCov-19 (Oxford–AstraZeneca) vaccine and 47% with the BNT162b2 (Pfizer–BioNTech) or the mRNA-1273 (Moderna) vaccine;^3^ all vaccinated children aged 12–17 years received one dose of the BNT162b2 (Pfizer–BioNTech) or mRNA-1273 (Moderna) vaccine.

In early September, 2021, children aged 5–17 years in England returned to school, with the Department for Education no longer recommending that it was necessary to keep children in consistent groups (referred to as social bubbles). Furthermore, schools were no longer expected to do contact tracing, with close contacts in schools now identified by the national contact tracing programme (the UK National Health Service [NHS] Test and Trace service).^4^ During this first month of children returning to school, the incidence of confirmed SARS-CoV-2 cases in England recorded through Pillar 2 dropped to a low in mid-September, 2021, and then rose again, but overall was relatively stable. This is in stark contrast to the situation in Scotland, where the incidence of confirmed SARS-CoV-2 cases increased by more than 450% during August, at a time when school-aged children (aged 5–17 years) in Scotland returned to school.^5^ Such national-level (and more local) trends are highly dependent on the context, including the local prevalence and dynamics of transmission of the delta variant, return to school, changes in social mixing patterns, home versus office working, and levels of vaccine-induced and naturally acquired immunity.

Here, we describe the underlying dynamics driving patterns in SARS-CoV-2 prevalence during September, 2021, in England by analysing RT-PCR swab positivity from the fourteenth round of data collection of the REal-time Assessment of Community Transmission-1 (REACT-1) study.^6^, ^7^ In this round, throat and nose swabs were obtained from a random sample of the population of England from Sept 9 to Sept 27, 2021. We also combined round 14 data with data from round 13, in which swabs were obtained from June 24 to July 12, 2021.

## Methods

### Study population

The REACT-1 study methods have been reported elsewhere.^7^ Briefly: with data collection starting May 1, 2020, we invited into the study random cross-sectional samples of the population in England (aged ≥5 years). Data were obtained monthly over a period of 2–3 weeks except for December, 2020, and August, 2021, when no survey was done. At each round of data collection, named individuals from the NHS list of patients registered with a general practitioner in England were invited to take part in the study, based on lists obtained from NHS Digital.

From May 1, 2020 (start of round 1), to May 3, 2021 (end of round 11), we aimed for approximately equal numbers of participants in each of the 315 lower-tier local authorities in England (combining the Isles of Scilly with Cornwall and the City of London with Westminster), but from round 12 (May 20 to June 7, 2021) onwards, we modified the sampling procedure to obtain a random sample in proportion to the population at the lower-tier local authority level. This increased the sampling in higher-population-density inner-city areas, although prevalence reporting was unaffected as we weighted the data at each round to be representative of England as a whole (described below).

### RT-PCR testing and vaccination data

Participants provided a self-administered throat and nose swab (or a parent or guardian obtained a swab for children aged 5–12 years) following written and video instructions. Swabs were sent to a central laboratory for analysis by RT-PCR. Extracted nucleic acid was analysed for the presence or absence of SARS-CoV-2 with the ViroBOAR 1·0 RT-qPCR kit (EuroFins Genomics, GmbH, Ebersberg, Germany) for SARS-CoV-2 on the Roche Lightcycler 480 II to detect in parallel two gene targets: the N gene and E gene. The assay has a specificity close to 100% and a limit of detection of ten copies per microlitre. Samples were considered positive either if the two gene targets (the N gene and E gene) were detected or if the N gene was detected with a cycle threshold (Ct) value less than 37.

For round 14, we modified the way that the swab samples were handled. From round 1 (starting May 1, 2020) to round 13 (ending July 12, 2021) we used dry swabs sent chilled to the laboratory by courier for RT-PCR testing. In round 14, we switched to wet swabs in saline solution, which were then allocated randomly on a 1:1 basis to either be sent by courier (n=46 705 valid swabs returned) or by post (n=53 822).

As part of the REACT-1 study, we obtained data on age, sex, address, and residential postcode from the NHS register, with further information on demographics, health (including self-reported vaccination status), and lifestyle derived from an online or telephone questionnaire. Participants were asked for consent for linkage to their NHS records, including data from the COVID-19 immunisation programme. Vaccination status from data linkage was derived with the assumption that one dose would only be effective from 14 days or more after injection. Unvaccinated people were therefore defined as those who had not received any vaccine dose or received one dose less than 14 days before swabbing; single-dose vaccinated people were defined as those who received one dose 14 days or more before swabbing and either no second dose or a second dose less than 14 days before swabbing; and people who had received two vaccine doses were defined as those who had received their second dose 14 days or more before swabbing.

### Viral genome sequencing

Subsequent to RT-PCR testing, positive samples with N gene Ct values less than 34 and with sufficient material were frozen and sent for viral genome sequencing at the Quadram Institute, Norwich, UK. The ARTIC SARS-CoV-2 sequencing protocol^8^ was used for amplification of viral RNA and the CoronaHiT platform used for preparation of sequencing libraries.^9^ The ARTIC bioinformatic pipeline^10^ was used for analysis of sequencing data, and lineages were assigned with PangoLEARN.^11^

### Statistical analyses

Statistical analyses were done in R software (R version 4.0.5 (31/03/2021, Shake and Throw). We calculated unweighted SARS-CoV-2 prevalence by sociodemographic, occupational, and other groups by dividing counts of swab positivity (from RT-PCR) by the number of valid swabs returned. We used rim weighting^12^ to obtain prevalence weighted to be representative of the population of England as a whole.

We used an exponential model of growth or decay to analyse trends in swab positivity over time. We assumed that the number of positive samples (from the total number of samples) each day arose from a binomial distribution based on the day of swabbing or, if unavailable, the day of sample collection (courier) or first scan of the sample by the Post Office, if sent by post. We estimated posterior credible intervals (CrIs) using a bivariate No-U-Turn Sampler assuming uniform prior distributions on the probability of swab positivity on day zero and the growth rate.^13^ We estimated the reproduction number (R) assuming generation time was gamma distributed with shape parameter n=2·29 and rate parameter β=0·36 (corresponding to a mean generation time of 6·29 days).^14^ We estimated R from the equation

$$\text{R}={\left(1+\frac{r}{\beta }\right)}^{n}$$

using data from all participants and stratified by age (5–17 years, 18–54 years, and ≥55 years).^15^ We also estimated R for different definitions of swab positivity, separately for samples sent by courier or post, and separately by age group and by region.

We fit a Bayesian penalised-spline (P-spline) model^16^ to the daily data using data from all rounds of REACT-1. Estimation of the daily prevalence of swab positivity used a No-U-Turn Sampler in logit space, with the data segmented into approximately 5-day sections by regularly spaced knots (providing an appropriate balance between time resolution and computational feasibility), and further knots included beyond the study period to minimise edge effects. We also fit P-splines to the REACT-1 data stratified by age as above, in which a P-spline was fit separately to each age group but the smoothing parameter, ρ, was assumed to be the best fitting value obtained for the model fit to all data. For clarity, although the estimates are based on the full REACT-1 dataset, here we only report estimates for collection days of round 14.

We estimated vaccine effectiveness against SARS-CoV-2 infection by combining data from rounds 13 and 14 to increase statistical power. We estimated vaccine effectiveness as 1 – odds ratio (OR), where the OR was obtained from a logistic regression model of the risk of swab positivity in double-vaccinated (ie, those with two doses, with the second dose administered more than 14 days before swabbing) and unvaccinated individuals, with adjustment for round, then additionally for age, and sex, and further adjustment for index of multiple deprivation quintile and ethnicity.

We obtained research ethics approval from the South Central-Berkshire B Research Ethics Committee (Integrated Research Application System [IRAS] ID: 283787).

### Role of the funding source

The funders had no role in the design and conduct of the study; data collection, data management, data analysis, and data interpretation; or in the preparation, review, or approval of this manuscript.

## Results

From 822 176 individuals invited to participate in round 14, 147 393 (17·9%) registered, and 100 527 (12·2%) provided a swab with a valid RT-PCR result, with 87 966 (87·5%) consenting to data linkage. In round 13, 841 227 participants were invited; 147 332 registered, and 98 233 (66·7%) of them provided a swab with a valid RT-PCR result. Pooling linked data from both rounds resulted in 172 862 participants, including 102 142 (49 923 in round 13 and 52 219 in round 14) aged 18–64 years who were included in our vaccine effectiveness analyses (appendix p 7). A description of the characteristics of REACT-1 participants from round 13 and round 14 showed that those agreeing to data linkage were more likely to be men, aged 45 years or older, not living in London, White, from a single-person household, and to have experienced COVID-19-related symptoms. Most of these differences were relatively small (appendix p 2). Prevalence was slightly higher and Ct values for positive samples slightly lower in swabs collected by courier than in those sent by post (appendix pp 3, 8).

**Figure 1:**
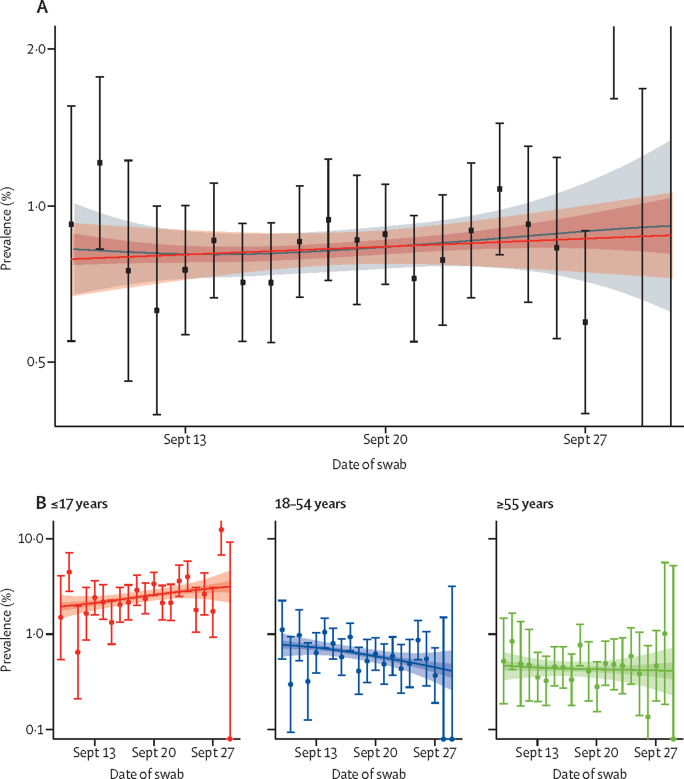
Weighted prevalence of swab positivity by day (A) Comparison of an exponential model fit to round 14 (red) and a P-spline model fit to data from all rounds of REACT-1 (grey). To improve readability, we only report P-spline estimates for sampling days in round 14 (Sept 9–27, 2021). The shaded red region shows the 95% posterior credible interval for the exponential model, and the shaded grey regions show the 50% (dark grey) and 95% (light grey) posterior credible interval for the P-spline model. Results are presented for each day (x-axis) of sampling for round 14 and the prevalence of swab positivity is shown (y-axis) on a log scale. Weighted observations (black dots) and 95% CIs (vertical lines) are also shown. (B) Comparison of P-spline models fit to all rounds of REACT-1 for participants aged 17 years and younger (red), those aged 18–54 years inclusive (blue), and those aged 55 years and older (green). Shown here only for the period of round 14. Shaded regions show 50% (dark shading) and 95% (light shading) posterior credible intervals for the P-spline models. Results are presented for each day (x-axis) of sampling for round 14 and the prevalence of swab positivity is shown (y-axis) on a log scale. Weighted observations (dots) and 95% CIs (vertical lines) are also shown.

Of the 100 527 valid swabs in round 14, 764 were positive, giving a prevalence of 0·83% (95% CrI 0·76–0·89) weighted to be representative of the population of England (appendix p 4). The highest weighted prevalence during September, 2021, was observed in teenagers aged 13–17 years, at 2·55% (95% CrI 2·11–3·08), and in children aged 5–12 years, at 2·32% (1·96–2·73; table 1, appendix p 9). The P-spline for round 14, fit to data from all REACT-1 rounds, was indicative of a stable or increasing trend in the prevalence of swab positivity (figure 1A), with R estimated at 1·03 (95% CrI 0·94–1·14) across all ages combined (table 2). The fitted P-splines indicated increasing prevalence at ages 5–11 years and decreasing prevalence at ages 18–54 years (figure 1B), with a greater than 99% posterior probability that the growth rate differed between these two groups. The corresponding R was 1·42 (95% CrI 1·18–1·68), with a greater than 0·99 posterior probability that R was greater than 1 at ages 5–11 years; R was 0·95 (0·76–1·16), with a posterior probability of 0·32 that R was greater than 1 in those aged 12–17 years; and R was 0·81 (0·68–0·97), with a posterior probability of 0·01 that R was greater than 1 in those aged 18–54 years (table 2).

**Table 1:** Unweighted and weighted prevalence of swab positivity in round 14

	**Positive swabs**	**Total swabs**	**Unweighted prevalence**	**Weighted prevalence**
**Sex**				
Female	428	55 976	0·76% (0·69–0·84)	0·83% (0·75–0·93)
Male	336	44 549	0·75% (0·68–0·84)	0·82% (0·73–0·92)
Unknown	0	2	0·00% (0·00–84·19)	··
**Age, years**				
5–12	155	6458	2·40% (2·04–2·80)	2·32% (1·96–2·73)
13–17	118	4927	2·40% (1·99–2·86)	2·55% (2·11–3·08)
18–24	9	2452	0·37% (0·17–0·70)	0·46% (0·23–0·90)
25–34	28	7374	0·38% (0·25–0·55)	0·36% (0·24–0·53)
35–44	100	12 118	0·83% (0·67–1·00)	0·79% (0·64–0·97)
45–54	130	16 855	0·77% (0·64–0·92)	0·78% (0·65–0·93)
55–64	113	20 856	0·54% (0·45–0·65)	0·55% (0·45–0·67)
65–74	80	19 313	0·41% (0·33–0·52)	0·42% (0·34–0·53)
≥75	31	10 174	0·30% (0·21–0·43)	0·29% (0·20–0·42)
**Region**				
South East	92	17 388	0·53% (0·43–0·65)	0·57% (0·45–0·72)
North East	39	4551	0·86% (0·61–1·17)	0·84% (0·60–1·18)
North West	121	12 117	1·00% (0·83–1·19)	0·99% (0·81–1·21)
Yorkshire and The Humber	105	9887	1·06% (0·87–1·28)	1·25% (1·00–1·57)
East Midlands	87	8830	0·99% (0·79–1·21)	1·15% (0·92–1·44)
West Midlands	88	10 249	0·86% (0·69–1·06)	1·01% (0·80–1·27)
East of England	87	11 756	0·74% (0·59–0·91)	0·73% (0·59–0·92)
London	91	14 885	0·61% (0·49–0·75)	0·62% (0·50–0·79)
South West	54	10 864	0·50% (0·37–0·65)	0·59% (0·43–0·80)
**Employment type**				
Health-care or care home worker	59	7963	0·74% (0·56–0·95)	0·80% (0·60–1·06)
Other essential or key worker	159	14 627	1·09% (0·93–1·27)	1·07% (0·90–1·28)
Other worker	263	38 496	0·68% (0·60–0·77)	0·71% (0·62–0·82)
Not full-time, part-time, or self-employed	244	37 312	0·65% (0·57–0·74)	0·77% (0·67–0·89)
Unknown	39	2129	1·83% (1·31–2·50)	1·88% (1·34–2·64)
**Ethnic group**				
White	635	87 942	0·72% (0·67–0·78)	0·78% (0·72–0·85)
Asian	58	5550	1·05% (0·79–1·35)	1·04% (0·77–1·41)
Black	22	1947	1·13% (0·71–1·71)	1·41% (0·91–2·19)
Mixed	18	1754	1·03% (0·61–1·62)	1·01% (0·62–1·63)
Other	12	1015	1·18% (0·61–2·06)	1·01% (0·56–1·82)
Unknown	19	2319	0·82% (0·49–1·28)	1·09% (0·67–1·75)
**Household size**				
1	57	16 613	0·34% (0·26–0·44)	0·33% (0·25–0·44)
2	174	39 044	0·45% (0·38–0·52)	0·46% (0·39–0·54)
3	150	17 235	0·87% (0·74–1·02)	0·93% (0·78–1·10)
4	250	19 154	1·31% (1·15–1·48)	1·32% (1·15–1·51)
5	89	6057	1·47% (1·18–1·81)	1·41% (1·12–1·76)
≥6	44	2424	1·82% (1·32–2·43)	1·75% (1·24–2·46)
**COVID-19 case contact**				
No	325	80 587	0·40% (0·36–0·45)	0·43% (0·38–0·49)
Yes, contact with a confirmed or tested COVID-19 case	293	4044	7·25% (6·47–8·09)	7·35% (6·50–8·31)
Yes, contact with a suspected COVID-19 case	38	1058	3·59% (2·55–4·90)	3·80% (2·70–5·32)
Unknown	108	14 838	0·73% (0·60–0·88)	0·79% (0·65–0·97)
**Symptom status**				
Classic COVID-19 symptoms	359	4963	7·23% (6·53–7·99)	6·85% (6·12–7·68)
Other symptoms	108	11 337	0·95% (0·78–1·15)	0·95% (0·77–1·17)
No symptoms	190	69 446	0·27% (0·24–0·32)	0·31% (0·27–0·37)
Unknown	107	14 781	0·72% (0·59–0·87)	0·79% (0·64–0·97)
**Number of children in the household**				
0	276	66 025	0·42% (0·37–0·47)	0·40% (0·35–0·46)
≥1	378	28 659	1·32% (1·19–1·46)	1·37% (1·22–1·52)
Unknown	110	5843	1·88% (1·55–2·26)	2·06% (1·69–2·51)
**Deprivation**				
1 (most deprived)	119	11 777	1·01% (0·84–1·21)	0·98% (0·81–1·20)
2	118	16 962	0·70% (0·58–0·83)	0·75% (0·61–0·92)
3	142	21 117	0·67% (0·57–0·79)	0·75% (0·63–0·90)
4	178	24 102	0·74% (0·63–0·85)	0·76% (0·65–0·89)
5 (least deprived)	207	26 569	0·78% (0·68–0·89)	0·90% (0·77–1·04)
**Vaccination status (self-reported)**				
Unknown	225	20 278	1·11% (0·97–1·26)	1·24% (1·07–1·42)
Unvaccinated	109	6969	1·56% (1·29–1·88)	1·73% (1·42–2·12)
Vaccinated: one dose	19	1542	1·23% (0·74–1·92)	1·41% (0·84–2·35)
Vaccinated: two doses*	386	67 332	0·57% (0·52–0·63)	0·56% (0·50–0·62)
Dose number not reported	25	4406	0·57% (0·37–0·84)	0·58% (0·38–0·88)
**Vaccination status linked data**†				
Unvaccinated	224	9467	2·37% (2·07–2·69)	2·34% (2·04–2·69)
Vaccinated: one dose	28	2208	1·27% (0·84–1·83)	1·29% (0·85–1·96)
Vaccinated: two doses	427	76 291	0·56% (0·51–0·62)	0·55% (0·49–0·61)

Data are point estimates (95% credible intervals for the weighted prevalence and 95% confidence intervals for unweighted prevalence), unless otherwise indicated.

* 177 participants who reported receiving three doses were included in the “Vaccinated: two doses” category.

† Linked data from round 14 included 87 966 participants, among whom 679 had positive swabs. Vaccination status for linked data was defined using time since last vaccination. We assumed that one dose would only be effective more than 14 days after injection. Unvaccinated individuals are defined as those who have not received any vaccine dose or who received one dose less than 14 days before swabbing; single-dose vaccinated individuals are defined as those who received one dose 14 days or more before swabbing, and either no second dose or a second dose within 14 days from testing; and double-dose vaccinated individuals are defined as those who received their second dose 14 days or more before swabbing.

**Table 2:** Growth rate and reproduction numbers from exponential model fit

		**Growth rate**	**R**	**Probability R>1**
All positive		0·005 (−0·010 to 0·021)	1·03 (0·94 to 1·14)	0·75
**Subset**				
	Positive for both E and N genes	0·009 (−0·007 to 0·024)	1·06 (0·96 to 1·16)	0·87
	Positive for both E and N genes or positive only for N gene with Ct 35 or less	0·007 (−0·008 to 0·023)	1·05 (0·95 to 1·15)	0·83
Shipment				
	Sent via post	0·009 (−0·011 to 0·030)	1·06 (0·93 to 1·20)	0·81
	Sent via courier	–0·003 (−0·026 to 0·020)	0·98 (0·84 to 1·13)	0·40
Age, years				
	5–11	0·059 (0·027 to 0·091)	1·42 (1·18 to 1·68)	>0·99
	12–17	–0·007 (−0·040 to 0·024)	0·95 (0·76 to 1·16)	0·32
	18–54	–0·031 (−0·057 to −0·005)	0·81 (0·68 to 0·97)	0·01
	≥55	–0·006 (−0·042 to 0·030)	0·96 (0·75 to 1·20)	0·37
	25–54 (no children in household)	–0·039 (−0·091 to 0·013)	0·77 (0·51 to 1·08)	0·07
	25–54 (children in household)	–0·014 (−0·045 to 0·019)	0·91 (0·74 to 1·12)	0·20
	25–54 (household size 1–2)	–0·025 (−0·080 to 0·032)	0·85 (0·56 to 1·21)	0·19
	25–54 (household size ≥3)	–0·018 (−0·050 to 0·013)	0·89 (0·71 to 1·08)	0·13
Region				
	East Midlands	0·052 (0·007 to 0·097)	1·36 (1·05 to 1·73)	0·99
	West Midlands	–0·016 (−0·059 to 0·028)	0·90 (0·66 to 1·18)	0·24
	East of England	0·024 (−0·023 to 0·071)	1·16 (0·86 to 1·51)	0·84
	London	0·080 (0·035 to 0·126)	1·59 (1·23 to 1·99)	>0·99
	North West	0·009 (−0·031 to 0·049)	1·06 (0·81 to 1·34)	0·67
	North East	–0·057 (−0·130 to 0·014)	0·67 (0·36 to 1·09)	0·06
	South East	–0·041 (−0·087 to 0·004)	0·76 (0·53 to 1·02)	0·04
	South West	0·003 (−0·054 to 0·060)	1·02 (0·69 to 1·42)	0·55
	Yorkshire and The Humber	–0·020 (−0·061 to 0·020)	0·87 (0·65 to 1·13)	0·16

Data are posterior median values and 95% credible intervals. The posterior probability that R is greater than 1 is also reported. Results are presented for the full population of REACT-1 round 14, for different definitions of swab positivity, different methods of sample shipment, and for models stratified by age or region.

Weighted prevalence also varied by region (table 1; appendix p 9), ranging from 0·57% (95% CrI 0·45–0·72) in the South East to 1·25% (1·00–1·57) in Yorkshire and The Humber. Within round 14, there was evidence of epidemic growth (with a posterior probability greater than 0·99 that R>1) in London, with an R of 1·59 (95% CrI 1·23–1·99), and in the East Midlands, with an R of 1·36 (1·05–1·73; table 2).

We found a higher weighted prevalence of swab positivity among participants of Black ethnicity, at 1·41% (95% CrI 0·91–2·19), compared with that of White participants, at 0·78% (0·72–0·85; table 1). Participants living in larger households had a higher weighted prevalence than those in smaller households, ranging from 0·33% (95% CrI 0·25–0·44) for single-person households to 1·75% (1·24–2·46) for households with six or more individuals (table 1). Prevalence was also higher in households with one or more children, at 1·37% (95% CrI 1·22–1·52), compared with 0·40% (0·35–0·46) for households without children; and weighted prevalence was 7·35% (6·50–8·31) among those who reported being in contact with a confirmed COVID-19 case, compared with 0·43% (0·38–0·49) among those without such contact (table 1).

Using self-reported vaccination status, we found a higher prevalence in unvaccinated participants (all ages), at 1·73% (95% CrI 1·42–2·12), compared with those reporting two vaccine doses, at 0·56% (0·50–0·62; table 1).

In multivariable logistic regression analysis, key workers other than health-care workers and care home workers had an increased risk of swab positivity compared with other workers (OR 1·35 [95% CI 1·10–1·66]; table 3). Swab positivity was also higher with increasing household size (mutually adjusted OR 1·77 [95% CI 1·44–2·17] for households of three to five people and 2·37 [1·62–3·47] for households of six or more people), compared to households with one or two people.

**Table 3:** Multiple logistic regression for participants (n=100 527) with valid swab test results in round 14

	**Adjusted for age and sex**	**Mutually adjusted**
**Sex**		
Male	Ref	Ref
Female	1·02 (0·88–1·18)	1·07 (0·92–1·24)
**Age group, years**		
5–12	2·96 (2·30–3·82)	2·65 (2·03–3·45)
13–17	2·95 (2·26–3·86)	2·77 (1·98–3·88)
18–24	0·44 (0·22–0·88)	0·45 (0·23–0·90)
25–34	0·46 (0·30–0·70)	0·52 (0·34–0·81)
35–44	Ref	Ref
45–54	0·93 (0·72–1·21)	0·98 (0·75–1·29)
55–64	0·66 (0·50–0·86)	0·84 (0·63–1·12)
≥65	0·46 (0·35–0·60)	0·74 (0·53–1·03)
**Region**		
North East	1·73 (1·18–2·51)	1·79 (1·21–2·65)
North West	1·95 (1·49–2·57)	2·00 (1·50–2·67)
Yorkshire and The Humber	2·03 (1·53–2·69)	2·08 (1·55–2·79)
East Midlands	1·93 (1·43–2·59)	1·92 (1·41–2·62)
West Midlands	1·63 (1·22–2·19)	1·73 (1·28–2·35)
East of England	1·40 (1·04–1·88)	1·31 (0·96–1·79)
London	1·08 (0·81–1·45)	1·12 (0·82–1·53)
South East	Ref	Ref
South West	0·99 (0·71–1·39)	1·00 (0·70–1·42)
**Key worker status**		
Health-care worker or care home worker	0·94 (0·71–1·25)	0·86 (0·64–1·16)
Key worker (other)	1·43 (1·17–1·75)	1·35 (1·10–1·66)
Other worker	Ref	Ref
Not full time, part-time, or self-employed	1·00 (0·81–1·24)	0·97 (0·79–1·20)
**Ethnicity**		
White	Ref	Ref
Asian	1·06 (0·81–1·40)	1·10 (0·82–1·48)
Black	1·16 (0·76–1·79)	1·28 (0·81–2·02)
Mixed	0·82 (0·51–1·33)	0·81 (0·48–1·36)
Other	1·30 (0·73–2·32)	1·48 (0·82–2·66)
**Household size**		
1–2 people	Ref	Ref
3–5 people	1·66 (1·36–2·02)	1·77 (1·44–2·17)
≥6 people	2·24 (1·57–3·20)	2·37 (1·62–3·47)
**Deprivation index quintile**		
1 (most deprived)	1·24 (0·98–1·55)	1·00 (0·78–1·29)
2	0·91 (0·72–1·14)	0·92 (0·72–1·16)
3	0·89 (0·72–1·11)	0·90 (0·72–1·13)
4	0·98 (0·80–1·20)	0·94 (0·76–1·16)
5 (least deprived)	Ref	Ref

Results are presented as odds ratios (ORs) and 95% CIs adjusted for age and sex and additionally for all other variables (mutually adjusted ORs for all variables shown).

**Figure 2:**
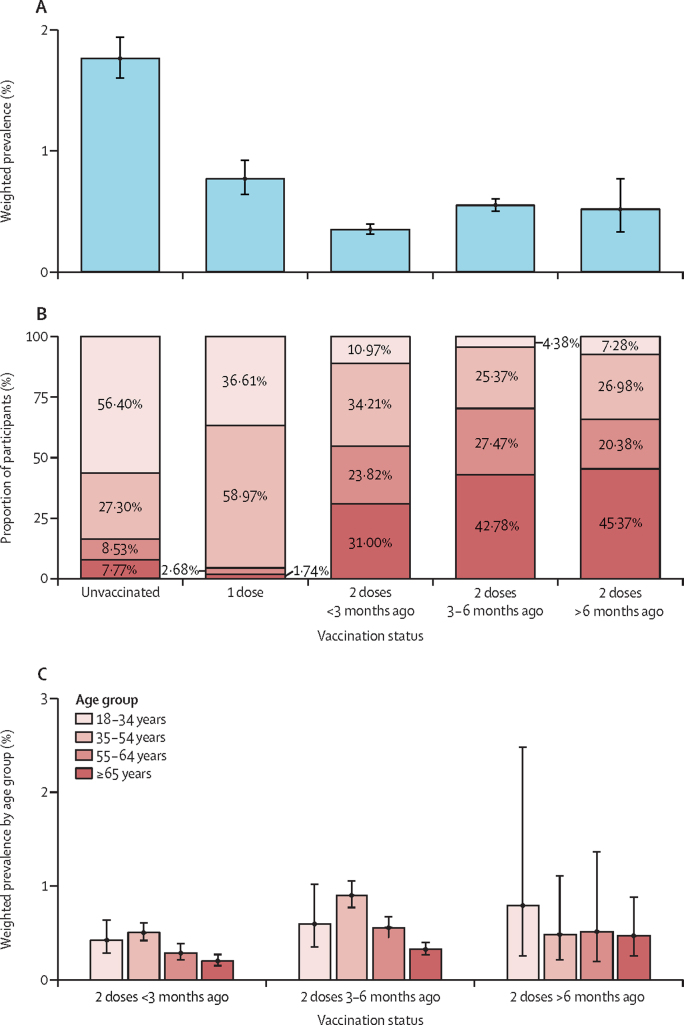
Weighted prevalence of swab positivity by vaccination status (A) Weighted prevalence of swab positivity for all REACT-1 participants aged 18 years and older with linked data in round 13 and round 14 combined by vaccination status (n=74 885 in round 13 and n=78 652 in round 14). (B) Age distribution within each vaccination status. (C) Weighted prevalence by age group in participants who received two vaccine doses (any vaccine).

We pooled the linked vaccine data from rounds 13 and 14 (172 862 participants overall, 19 325 aged <18 years, 102 142 aged 18–64 years, and 51 395 aged ≥65 years). Because of the relatively small numbers of individuals vaccinated below the age of 18 years, corresponding participants (n=19 325) were excluded from our vaccine effectiveness analyses. Weighted prevalence was higher for those who received their second dose 3–6 months before their swab than for those whose second dose was up to 3 months before their swab (0·55% [95% CrI 0·50–0·61] *vs* 0·35% [0·31–0·40]; figure 2A; appendix p 5). The weighted prevalence for individuals whose second dose was administered more than 6 months before their swab was similar to that of those vaccinated within 3–6 months of their swab, at 0·52% (95% CrI 0·33–0·78), but with a wider credible interval. The age distribution of participants by vaccination status indicated, as expected from the age-based rollout of the vaccine programme in England, a higher proportion of individuals aged 35 years and older among those who had received two vaccine doses compared to those who had received a single dose, or those who were unvaccinated (appendix p 5). Nevertheless, among those who had received two vaccine doses, we observed similar age distributions in people who had received their second dose 3–6 months or more than 6 months before swabbing. Regardless, in individuals aged 18 years and older with linked data on vaccination status, weighted prevalence in unvaccinated people, at 1·76% (95% CrI 1·60–1·95), was three to five times higher than in double-vaccinated individuals, at 0·47% (0·43–0·51). Reflecting the rollout of the vaccine programme in England, a higher proportion of individuals who were unvaccinated or who had a single dose of the vaccine were younger (≤54 years) than those with two doses (figure 2B; appendix p 5). However, in double-vaccinated participants, there was an apparent trend of increasing weighted prevalence in each age group for those who had received their second dose 3–6 months before their swab compared to those who were vaccinated up to 3 months before (figure 2C).

Vaccine effectiveness estimates were derived with the linked dataset for rounds 13 and 14 combined for individuals aged 18–64 years only, since few people older than 65 years were unvaccinated (1·1%), and, as noted, few people younger than 18 years were vaccinated (6·3% in rounds 13 and 14 combined). For all individuals aged 18–64 years and all vaccines combined, vaccine effectiveness against infection was 66·3% (95% CI 55·3–74·7), with adjustment for round, and 62·8% (49·3–72·7) in the fully adjusted model. Fully adjusted vaccine effectiveness point estimates for the BNT162b2 (Pfizer–BioNTech) and mRNA-1273 (Moderna) vaccines were comparable (71·3% *vs* 75·1%, with a much wider confidence interval for the mRNA-1273 [Moderna] vaccine due to a smaller number of observations), and higher than that estimated for the ChAdOx1 nCov-19 (Oxford–AstraZeneca) vaccine (44·8%). However, differences in vaccine effectiveness estimates by vaccine type did not reach statistical significance at a 0·05 significance level. Inclusion of an interaction term for vaccine by round was not suggestive of effects being modified across rounds 13 and 14 (data not shown). Vaccine effectiveness estimates in models restricted to symptomatic cases were similar to those from models that also included asymptomatic cases (table 4).

**Table 4:** Mean vaccine effectiveness against infection for rounds 13 and 14* of REACT-1

	**Test negatives (%)**			**Test positives (%)**			**Vaccine effectiveness (95% CI)**	**Interaction p value**†
	Round 13	Round 14	Total	Round 13	Round 14	Total		
**Full population**								
Unvaccinated	2907 (73·9%)	1026 (26·1%)	3933	44 (77·2%)	13 (22·8%)	57	··	··
**All vaccines**								
Round	34 853 (41·2%)	49 688 (58·8%)	84 541	144 (30·5%)	328 (69·5%)	472	66·3% (55·3–74·7)	0·05
Round, age, and sex	34 853 (41·2%)	49 688 (58·8%)	84 541	144 (30·5%)	328 (69·5%)	472	61·4% (47·5–71·6)	0·11
Round, age, sex, IMD, region, and ethnicity	34 853 (41·2%)	49 688 (58·8%)	84 541	144 (30·5%)	328 (69·5%)	472	62·8% (49·3–72·7)	0·09
**AstraZeneca vaccine**								
Round	24 934 (44·3%)	31 290 (55·7%)	56 224	109 (30·5%)	248 (69·5%)	357	62·3% (49·7–71·8)	0·02
Round, age, and sex	24 934 (44·3%)	31 290 (55·7%)	56 224	109 (30·5%)	248 (69·5%)	357	41·8% (18·5–58·4)	0·13
Round, age, sex, IMD, region, and ethnicity	24 934 (44·3%)	31 290 (55·7%)	56 224	109 (30·5%)	248 (69·5%)	357	44·8% (22·5–60·7)	0·11
**Pfizer–BioNTech vaccine**								
Round	6500 (33·5%)	12 888 (66·5%)	19 388	26 (31·7%)	56 (68·3%)	82	70·7% (57·5–79·7)	0·51
Round, age, and sex	6500 (33·5%)	12 888 (66·5%)	19 388	26 (31·7%)	56 (68·3%)	82	70·0% (55·0–80·0)	0·56
Round, age, sex, IMD, region, and ethnicity	6500 (33·5%)	12 888 (66·5%)	19 388	26 (31·7%)	56 (68·3%)	82	71·3% (56·6–81·0)	0·49
**Moderna vaccine**								
Round	24 (2%)	1 197 (98·0%)	1 221	0 (0·0%)	4 (100·0%)	4	74·4% (21·9–91·6)	0·98
Round, age, and sex	24 (2%)	1 197 (98·0%)	1 221	0 (0·0%)	4 (100·0%)	4	76·4% (27·6–92·3)	0·98
Round, age, sex, IMD, region, and ethnicity	24 (2%)	1 197 (98·0%)	1 221	0 (0·0%)	4 (100·0%)	4	75·1% (22·7–92·0)	0·98
**Unknown**								
Round	3395 (44·0%)	4313 (56·0%)	7 708	9 (31·0%)	20 (69·0%)	29	74·9% (59·8–84·3)	0·15
Round, age, and sex	3395 (44·0%)	4313 (56·0%)	7 708	9 (31·0%)	20 (69·0%)	29	68·1% (46·6–81·0)	0·28
Round, age, sex, IMD, region, and ethnicity	3395 (44·0%)	4313 (56·0%)	7 708	9 (31·0%)	20 (69·0%)	29	67·1% (44·1–80·6)	0·16
**Symptomatic positives only**‡								
Unvaccinated	2907 (73·9%)	1026 (26·1%)	3 933	44 (77·2%)	13 (22·8%)	77	··	··
**All vaccines**								
Round	34 853 (41·2%)	49 688 (58·8%)	84 541	64 (22·2%)	224 (77·8%)	288	67·8% (53·0–77·9)	0·03
Round, age, and sex	34 853 (41·2%)	49 688 (58·8%)	84 541	64 (22·2%)	224 (77·8%)	288	64·4% (46·8–76·2)	0·04
Round, age, sex, IMD, region, and ethnicity	34 853 (41·2%)	49 688 (58·8%)	84 541	64 (22·2%)	224 (77·8%)	288	66·4% (49·6–77·6)	0·04
**AstraZeneca vaccine**								
Round	24 934 (44·3%)	31 290 (55·7%)	56 224	49 (21·7%)	177 (78·3%)	226	62·3% (44·9–74·3)	0·01
Round, age, and sex	24 934 (44·3%)	31 290 (55·7%)	56 224	49 (21·7%)	177 (78·3%)	226	42·4% (11·0–62·7)	0·05
Round, age, sex, IMD, region, and ethnicity	24 934 (44·3%)	31 290 (55·7%)	56 224	49 (21·7%)	177 (78·3%)	226	45·5% (15·5–64·9)	0·06
**Pfizer–BioNTech vaccine**								
Round	6500 (33·5%)	12 888 (66·5%)	19 388	9 (21·4%)	33 (78·6%)	42	76·5% (61·1–85·8)	0·23
Round, age, and sex	6500 (33·5%)	12 888 (66·5%)	19 388	9 (21·4%)	33 (78·6%)	42	74·2% (55·4–85·1)	0·34
Round, age, sex, IMD, region, and ethnicity	6500 (33·5%)	12 888 (66·5%)	19 388	9 (21·4%)	33 (78·6%)	42	76·6% (59·0–86·6)	0·32
**Moderna vaccine**§								
Round	24 (2%)	1197 (98%)	1 221	0	0	0	··	··
**Unknown**								
Round	3395 (44·0%)	4313 (56·0%)	7708	6 (30·0%)	14 (70·0%)	20	70·2% (46·3–83·4)	0·30
Round, age, and sex	3395 (44·0%)	4313 (56·0%)	7708	6 (30·0%)	14 (70·0%)	20	59·9% (23·2–79·1)	0·53
Round, age, sex, IMD, region, and ethnicity	3395 (44·0%)	4313 (56·0%)	7708	6 (30·0%)	14 (70·0%)	20	61·1% (23·7–80·2)	0·36

Data are means (95% CIs), and adjusted for round and further adjusted for age and sex, and index of multiple deprivation (IMD), region, and ethnicity for participants aged 18–64 years and those reporting at least one symptom in month before swabbing.

* Of the participants aged 18–64 years with valid swabs from round 13 (n=57 457) and round 14 (59 655), 49 923 consented to have their data linked in round 13, as did 52 219 in round 14.

† p value for the multiplicative interaction term assessing possible modification of the effect of vaccination status by round.

‡ Symptomatic positive individuals are defined as those with a positive swab having reported at least one of 29 surveyed symptoms in the month before swabbing.

§ No symptomatic positive swab was observed in rounds 13 and 14; we therefore did not calculate vaccine effectiveness in this instance.

Lineages from the 475 (62·2%) samples sequenced among the 764 positive swabs were all of the delta variant or sublineages of delta. AY.4 was the most detected sublineage, representing 61·5% (95% CI 57·0–65·7) of the sequenced samples, and there were seven samples with single spike mutations of interest as defined by Public Health England (now the UK Health Security Agency): Glu484Lys, Asn501Tyr, Phe490Ser, Arg246Gly, Val483Phe, Pro251Leu, and Gln613His (appendix p 6). We identified 22 samples (eight with the B.1.617.2 lineage and 14 the AY.4 lineage) including the Tyr145His mutation in the spike protein, associated with the AY.4.2 delta sublineage, corresponding to 4·63% (95% CI 3·07–6·91) of all lineages determined.

## Discussion

In this fourteenth round of the REACT-1 study, we found both high and increasing prevalence of SARS-CoV-2 swab positivity among school-aged children in England during September, 2021, reflecting increased social mixing of children as they attended school for the autumn term. At the same time, we found decreasing prevalence among young to middle-aged adults (18–54 years). This observation might reflect the effect of previous natural infection, especially among younger adults in whom infection rates have been high,^17^ and the continued rollout of the vaccination programme in England. From April, 2021, the national COVID-19 vaccination programme was expanded to include adults younger than 50 years,^18^ a single vaccine dose for older teenagers (aged 16–17 years) from August, 2021, and a single dose for children aged 12–15 years from mid-September, 2021. As of mid-to-late December, 2021, all children aged 12 years and older are being offered a second vaccine dose, and a third (booster) dose is being rolled out to the adult population aged 18 years and older.

As in our previous report^19^ (round 13), we found that all sequenced swabs were of the delta variant and its sublineages, indicating almost complete replacement of alpha and other variants by delta in England at that time. We detected one potential escape Glu484Lys mutation, which translates into an estimated 984 such infections in England with a lower 95% confidence limit of 159. Overall, there were seven samples with single spike mutations of interest, as defined by Public Health England. An additional 22 samples included the Tyr145His mutation in the spike protein associated with the AY.4.2 sublineage. The corresponding AY.4 proportion of 4·63% (95% CI 3·07–6·91) across all lineages is in line with the 6% of cases reported by Public Health England in the week commencing Sept 27, 2021.^20^

Vaccination has proved highly effective against severe complications of COVID-19, including hospital admissions and death, but there is less clarity concerning protection against infection. Although estimates of vaccine effectiveness against infection of up to 90% have been reported, based on routine testing of symptomatic individuals,^21^ here we report an estimate of vaccine effectiveness against infection of 63% from REACT-1 rounds 13 and 14, when the delta variant dominated. This finding is similar to what was reported in the linked data for round 13 alone,^19^ but by combining data from both rounds 13 and 14 we were also able to estimate vaccine effectiveness for the ChAdOx1 nCov-19 (Oxford–AstraZeneca) and BNT162b2 (Pfizer–BioNTech) vaccines separately. Although the confidence intervals overlap, our results suggest higher effectiveness against infection with the BNT162b2 (Pfizer–BioNTech) vaccine than with the ChAdOx1 nCov-19 (Oxford–AstraZeneca) vaccine. Greater differences between vaccines were reported in a previous study,^22^ and by Public Health England (now UK Health Security Agency [UK HSA]) based on routine testing of symptomatic cases.^23^ Nonetheless, it should be noted that vaccine effectiveness is specific to population and time so these estimates reflect the performance of the vaccines in England during a specific time period (ie, June–September, 2021). Since then, the omicron (B.1.1.529) variant had become dominant in England by December, 2021, with studies by UK HSA indicating lower vaccine effectiveness against symptomatic infection for omicron compared to delta.^24^

Our study shows that the prevalence of swab positivity among unvaccinated individuals (including all children aged 5–11 years) or individuals who received one vaccine dose (including children aged 12–17 years) was three to four times higher than in double-vaccinated people, but also suggests that the prevalence of swab positivity indicative of breakthrough infections following two-dose vaccination might increase after 3–6 months. Additionally, we found that people living in larger households, and people living in households with children, had higher rates of swab positivity than those in smaller households or those without any children in the household.

The finding of the highest weighted prevalence levels in swab positivity during round 14 in children aged 5–11 years and 12–17 years raises concerns for clinically extremely vulnerable children as well as clinically extremely vulnerable close contacts, including household members and school staff. As of the end of December, 2021, clinically vulnerable children aged 5–11 years will be offered two doses of vaccine. There are also concerns about the effects on education for the large numbers of children who are required to be out of school when testing positive as a result of the high rates of SARS-CoV-2 infection. The finding that prevalence was more than three times greater for individuals in households with one or more children than in individuals in households without children suggests that infections in children, unsurprisingly, spread into other age groups. Higher viral loads associated with the delta variant in younger individuals compared to earlier variants^25^, ^26^, ^27^ might also increase the transmission efficiency of children and therefore contribute to higher within-household infections.

As of Sept 27, 2021, only 1214 (6·3%) children aged 12–17 years had been vaccinated in the REACT-1 study, thus not allowing a meaningful extension of our vaccine effectiveness analyses to that age group in round 14. Further studies of vaccine effectiveness are warranted given the rapid increase in omicron infections in England beginning in December, 2021, and the rollout of double-dose vaccinations to school children aged 12–15 years, booster doses at ages 16–17 years and in adults aged 18 years and older. Additionally, community-based studies such as REACT-1 enable the estimation of vaccine effectiveness against infection among those with asymptomatic as well as symptomatic infections.

Although following the vaccination campaign there has been a relative uncoupling between SARS-CoV-2 infections and hospital admissions and deaths in England,^2^ concerns remain about the potential for high infection rates and incomplete population immunity to result in an increased risk of severe complications from COVID-19. England, in common with the rest of the UK and several other countries (notably Israel^28^), embarked on a campaign to roll out third (booster) doses—from October, 2021, in England. This initially involved prioritisation of adults aged 50 years and older, health and social care workers, and younger people at risk, but rollout was subsequently extended to all adults aged 18 years and older as of the end of December, 2021.^29^ Ongoing monitoring of the epidemic in England and elsewhere will be important to gauge the extent to which booster doses in adults and vaccination in children curtail transmission of new SARS-CoV-2 variants of concern such as the omicron variant.

Our study has limitations. Since the REACT-1 study began data collection in May, 2020, we observed a gradual reduction in response rates, from 30·5% in round 1 to 11·7% in round 13. However, in round 14, the response rate increased slightly to 12·2%. This increase, albeit small, is encouraging. It might be that further changes to the survey could further increase participation. The change to using wet swabs in saline solution and collection of samples without the cold chain (by post or courier) might have affected diagnostic sensitivity. We were reassured by the limited differences between the samples collected by post and courier. However, because the exact system used in previous rounds (dry swabs and a sustained cold chain) was not compared within a round, we could not estimate the impact of the new approach compared to that used in previous rounds. A further limitation is that we do not have accurate data on the vaccination status of all participants. Although consent for data linkage was at a high level (87·5% in round 14), not all participants consented for linkage to their NHS records, which include data from the COVID-19 immunisation programme. For those whose data are not linked, data on the dates of vaccination and vaccine type are either missing or less reliable than in the linked data; therefore, we based our estimates of vaccine effectiveness on the subset of participants with linked records. This approach might have introduced a bias to the extent that those who do and do not consent to data linkage might differ in important ways, such as social mixing patterns, that might affect the risk of infection. Additionally, for our estimates of vaccine effectiveness by vaccine type, even though we controlled for age and round in our sample, the amplitude of the differences between vaccines might be exaggerated. This is because there were different age-specific patterns in vaccine delivery (with the ChAdOx1 nCov-19 [Oxford–AstraZeneca] vaccine having been primarily administered to people aged ≥40 years) as well as differences in transmission dynamics for the age groups that received the different vaccines.

REACT-1 was not in the field during August, 2021, so there was a delay of nearly 2 months between the end of round 13 on July 12, and the beginning of round 14 on Sept 9, 2021. Although it is possible that vaccine effectiveness estimates could vary over this period, we did not find evidence for an interaction between round and vaccine effects.

In conclusion, we found evidence of increasing prevalence of swab positivity among school-aged children as well as higher prevalence of swab positivity 3–6 months following two-dose vaccination against COVID-19 in adults. Ongoing efforts to vaccinate school-aged children and deliver a booster dose to all adults aged 18 years and older should help to counteract any reduction in immunity at both individual and population levels. In the winter season in England, the NHS typically comes under strain from influenza and other viruses, and this has been exacerbated during December, 2021, by the rapid increase in omicron infections. It is therefore important that the vaccination programme maintains high coverage, including boosters, and reaches high proportions of children and unvaccinated or partially vaccinated adults to reduce SARS-CoV-2 transmission and the associated disruptions to work and education.

## Data sharing

Access to REACT-1 data are restricted because of ethical and security considerations. Summary statistics and descriptive tables from the current REACT-1 study are available in the appendix and online. Data from each round of the REACT-1 programme are summarised online. Additional summary statistics and results from the REACT-1 programme are also available online. REACT-1 study materials are also available for each round online.
